# COVID-19 in cancer patients with diabetes in Pakistan: Clinical features and management

**DOI:** 10.3389/fonc.2022.922579

**Published:** 2022-08-18

**Authors:** Kashif Asghar, Muhammad Abu Bakar, Sara Ashfaq, Asim Munir Alvi, Waqas Shafiq, Umal Azmat, Ahmed Imran Siddiqi, Asim Farooq, Rabail Raza, Kashif Siddique

**Affiliations:** ^1^ Department of Basic Sciences Research, Shaukat Khanum Memorial Cancer Hospital and Research Centre, Lahore, Pakistan; ^2^ Department of Cancer Registry and Clinical Data Management, Shaukat Khanum Memorial Cancer Hospital and Research Centre, Lahore, Pakistan; ^3^ Department of Endocrinology, Shaukat Khanum Memorial Cancer Hospital and Research Centre, Lahore, Pakistan; ^4^ Department of Clinical Research, Shaukat Khanum Memorial Cancer Hospital and Research Centre, Lahore, Pakistan; ^5^ Department of Radiology, Shaukat Khanum Memorial Cancer Hospital and Research Centre, Lahore, Pakistan

**Keywords:** cancer, diabetes, survivors, non-survivors, Pakistan, COVID-19

## Abstract

**Background:**

Diabetes and cancer are the leading causes of mortality all over the world. Infectious diseases are more common and/or life-threatening in patients with diabetes. Cancer patients with diabetes are individuals that are more susceptible to the current COVID-19 pandemic. We investigated the clinical features of survivor and non-survivor COVID-19-infected cancer patients with diabetes.

**Patients and Methods:**

We did a retrospective study of 43 diabetic cancer patients with PCR-confirmed COVID-19 infection from Shaukat Khanum Memorial Cancer Hospital and Research Centre, Lahore, Pakistan between March 03, 2020, and May 18, 2021. These patients either were discharged from the hospital or had died by Jun 16, 2021. Clinicopathological and radiological features were compared between survivors and non-survivors by fisher’s exact test and chi-square test.

**Results:**

Forty-three diabetic cancer patients with SARS-CoV-2 infection were enrolled and the majority were males 26 (60.5%). The overall mean age was 61.67 ± 11.80. 39 (90.7%) had solid tumors and 3 (7.0%) had hematological malignancies. Fever (74.4%) and dyspnea (58.1%) were the most common symptoms. Complications were reported in 36 (83.7%) patients; during the course of the disease. Additionally, all the deceased patients (n=15) had acquired the complications. 11 (25.6%) patients were admitted to an intensive care unit (ICU). Furthermore, 29 (67.4%) out of 43 patients showed abnormal features in the radiological findings. We found significantly elevated levels of C-reactive protein (*P*=0.005), serum lactate (*P*=0.01), albumin (*P*=0.02), alkaline phosphate (*P*=0.03), and neutrophil count (*P*=0.04) in the non-survivors as compared to the survivors.

**Conclusion:**

Cancer patients with diabetes are a vulnerable population in the current pandemic. Identifying how diabetes in cancer patients affects the severity of SARS-CoV-2 infection is crucial for the clinical management of these patients. Rigorous scrutiny of clinicopathological features of COVID-19 infected cancer patients with diabetes especially values of C-reactive protein, lactate, albumin, alkaline phosphate, neutrophils, and regular monitoring of blood glucose levels may play a critical role in the outcome of the disease.

## Introduction

In 2019, Wuhan City, China underwent an outbreak of respiratory disease caused by a novel coronavirus SARS-Cov-2 (1). The disease was consequently named COVID-19 (2, 3).** **The whole world including Pakistan experienced COVID-19 outbreaks with a devastating force (4, 5). It has been observed that the severity and fatality of COVID-19 are enhanced in the elderly or in patients with underlying comorbidities, specifically cancer, diabetes, cardiovascular diseases, lung and renal diseases (6–9).

It is estimated that Pakistan had 178,388 new cancer cases in the year 2020 (10). Shaukat Khanum Memorial Cancer Hospital and Research Centre (SKMCH & RC) is a state-of-the-art charitable cancer hospital in Lahore, Pakistan  (4, 11). Several studies demonstrated that cancer patients infected with COVID-19 had poorer prognoses and outcomes (12–14). We published the first comprehensive data of COVID-19 infected cancer patients from Pakistan and showed that these patients are a high-risk population with an increased mortality rate (15).

Diabetes and cancer are the leading causes of mortality all over the world (16, 17). The susceptibility of diabetic patients to contract the infection is still obscure, but it is definite that once they are infected, the patients are at high risk for severe disease (16). Moreover, it has also been established that the overall survival of diabetic patients, who develop cancer, is worse as compared to non-diabetic patients (17).

We investigated the data from diabetic cancer patients with SARS-CoV-2 infection who were admitted at SKMCH&RC. We analyzed the hematological, pathological, and radiological parameters in these patients. Here, we report the clinical characteristics of survivors and non-survivors of COVID-19-infected diabetic cancer patients in Pakistan, in an attempt to identify the risk factors associated with severe disease in this group of patients.

## Methods

### Study design and participants

This is a retrospective cohort study. After the COVID-19 outbreak, SKMCH & RC initiated the diagnosis and treatment of SARS-CoV-2 infected cancer patients based on the guidelines provided by WHO (18). All patients (aged ≥ 18 years) included in the current study had a history of cancer and diabetes. The patients had a diagnosis of either solid or hematological malignancies along with PCR confirmation of SARS-CoV-2 infection. We investigated the clinical features of survivors and non-survivors of COVID-19 infected diabetic cancer patients from Pakistan. The study cutoff date was May 18, 2021. The institutional review board (IRB) of SKMCH & RC approved this study (IRB-EX-20-04-20-01). IRB permitted the waiver of the written informed consent from participants.

### Data collection

Demographic data, clinicopathological and radiological characteristics were acquired from the medical records system of SKMCH & RC. Data about gender, age, comorbidities, cancer history, vital signs, body mass index and, symptoms and pathology lab tests were all obtained at the time of diagnosis of COVID-19 in cancer patients. According to the TNM staging system, cancers were defined as stages I, II, III, and IV. We also collected the data about therapies provided to diabetic cancer patients with COVID-19, complications, and outcomes during admission to the hospital.

### Statistical analysis

We hypothesized that differences exist in demographic, clinical, and laboratory characteristics, treatments, and cancer history between survivors and non-survivors of COVID-19-infected diabetic cancer patients. Quantitative variables were presented as medians/mean (range: minimum-maximum/standard deviation), and qualitative variables were presented by frequencies and percentages. The Mann-Whitney U test, Fisher’s exact test, and Chi-square test were applied to analyze the differences between groups according to the type of data. Kaplan-Meier analysis was used to check the overall survival. The differences between groups were considered to be significant when the P-value was less than 0.05.

## Results

**Figure 1:**
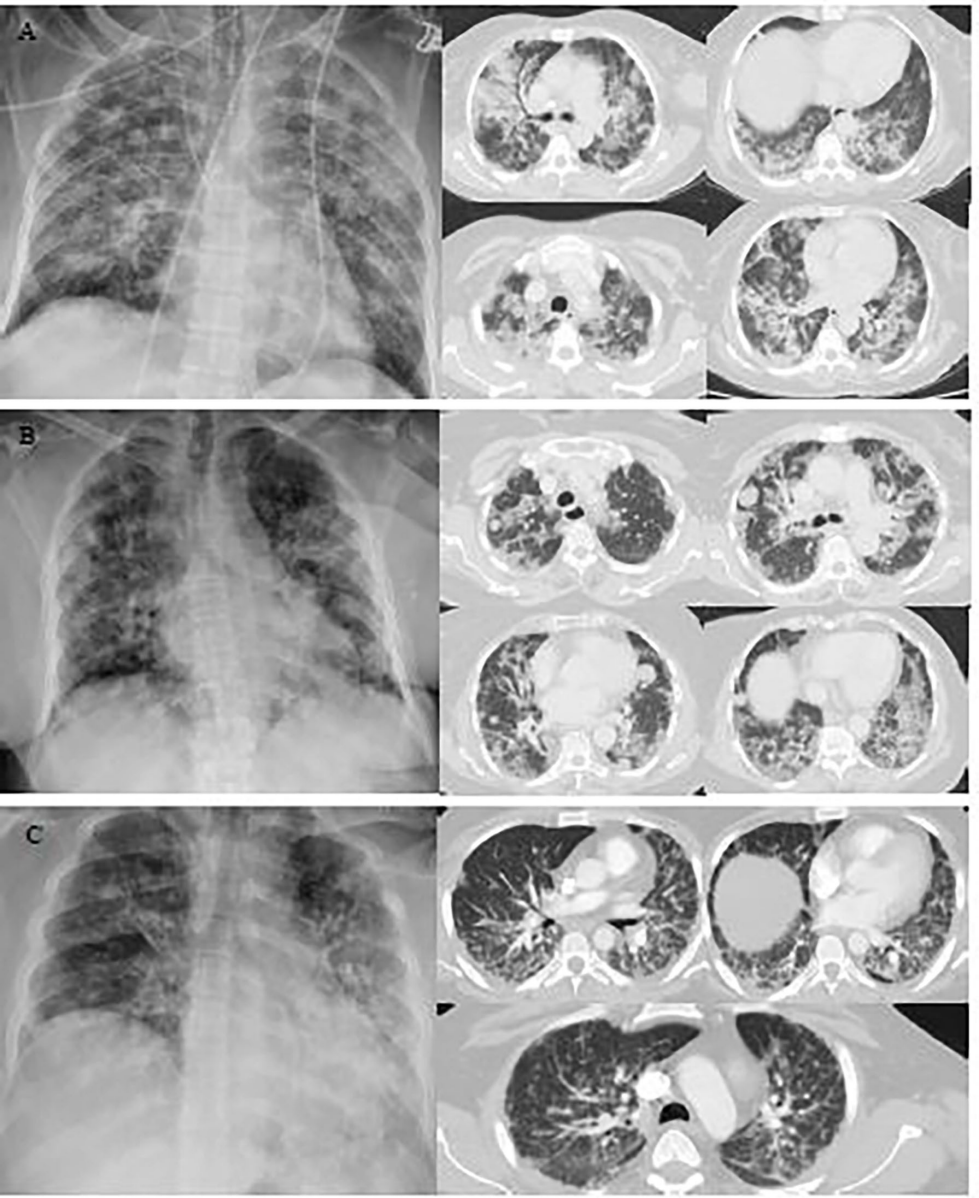
Representative images of the chest X-ray and chest computed tomography (CT) scan of COVID-19 cancer patients with diabetes. **(A)** A chest X-ray image of a 65-year-old female patient with scattered patchy airspace changes with prominent interstitial markings in bilateral lungs. Bilateral diffuse confluent airspace consolidations with COVID pneumonia. **(B)** Chest X-ray image of a 64-year-old female patient with interval increase in reticulation and interstitial lung disease with pulmonary nodules. Multifocal pulmonary metastases on the background of severe COVID pneumonia. **(C)** Chest X-ray image of a 40-year-old male patient with bilateral air space opacification, relatively confluent in the left lung. Peribronchial inflammatory cuffing with thickening of the intralobular septa with extensive pulmonary changes of COVID 19 infection seen bilaterally.

We retrospectively enrolled 43 cancer patients with diabetes and PCR-confirmed COVID-19 infection admitted to SKMCH & RC, Lahore, between March 03, 2020, and May 18, 2021. None of the 43 patients was lost to follow-up during the hospital admission. Of the 43 patients, 15 (34.9%) had died as of Jun 16, 2021. The main cause of death was SARS-CoV-2 infection in these patients. 26 (60.5%) patients were male, 17 (39.5%) patients were female, and the overall follow-up for all the patients was 6 days. The overall mean age was 61.67 ± 11.80 (**Table 1**). 32.56% of patients were admitted to the hospital within 24 hours after diagnosis. 20 (46.51%) of 43 patients had symptoms during the course of COVID-19. Fever (74.4%), dyspnea (58.1%), cough (41.9%) and muscle ache (32.6%) were the most common symptoms (**Table 1**). The cancer patients with diabetes also had other comorbidities (**Table 1**). Non-survivor patients had higher pulse rates (108.13± 25.71) and lower levels of blood oxygen saturation (SpO2) (88.20± 9.12) as compared to the survivors. There was a statistically significant mean difference in body mass index (BMI) versus survival and non-survival (*P*=0.001). Additionally, in non-survivor patients, 4 (27%) had underweight BMI. No significant differences in gender, age, and other comorbidities were identified among survivors and non-survivors groups. We found significantly elevated levels of C-reactive protein in the non-survivors as compared to the survivors (*P*=0.005). Furthermore, the non-survivor patients presented with higher levels of neutrophils (*P*=0.04) and alkaline phosphate (*P*=0.03), serum lactate (*P*=0.01), and albumin (*P*=0.02) (**Table 2**). Moreover, 29 (67.4%) out of 43 patients showed abnormal features in the radiological findings (**Figure 1**). 20 (46.5%) of 43 patients presented with consolidation (**Table 2**).

**Table 1:** Demographics and baseline characteristics of cancer patients with diabetes and COVID-19.

	Total (n = 43)	Alive 28 (65.1%)	Dead 15 (34.9%)	*P*-value
**Demographics**				
**Age (years)**				0.71
Mean ± SD	61.67 ± 11.80	62.18 ± 11.14	60.73 ± 13.30	
**Sex**				0.96
Male	26 (60.5%)	17 (60.7)	9 (60.0)	
Female	17 (39.5%)	11 (39.3)	6 (40.0)	
**Location**				0.66
Punjab	37 (86.0)	25 (89.3)	12 (80.0)	
FATA	2 (4.7)	1 (3.6)	1 (6.7)	
Khyber Pakhtunkhwa	3 (7.0)	2 (7.1)	1 (6.7)	
Balochistan	1 (2.3)	–	1 (6.7)	
**Clinical characteristics and outcomes**				
**Comorbidities**				
Coronary heart disease	3 (7.0)	2 (7.1)	1 (6.7)	1.00
Hypertension	11 (25.6)	9 (32.1)	2 (13.3)	0.27
Chronic pulmonary disease	2 (4.7)	2 (7.1)	–	0.53
Chronic kidney disease	3 (7.0)	1 (3.6)	2 (13.3)	0.27
Chronic liver disease	1 (2.3)	1 (3.6)	–	1.00
**Symptoms**				
Fever	32 (74.4)	23 (82.1)	9 (60.0)	0.15
Cough	18 (41.9)	12 (42.9)	6 (40.0)	1.00
Sore throat	5 (11.6)	2 (7.1)	3 (20.0)	0.32
Dyspnea	25 (58.1)	17 (60.7)	8 (53.3)	0.75
Chills	9 (20.9)	7 (25.0)	2 (13.3)	0.46
Headache	2 (4.7)	1 (3.6)	1 (6.7)	1.00
Muscle ache	14 (32.6)	10 (35.7)	4 (26.7)	0.83
Vomiting	3 (7.0)	–	3 (20.0)	0.04
Abdominal pain	1 (2.3)	1 (3.6)	–	1.00
Diarrhea	4 (9.3)	2 (7.1)	2 (13.3)	0.74
**Temperature**				
Mean ± SD	37.40 ± 0.97	37.39 ± 1.10	37.40 ± 0.74	0.98
**Pulse rate**				
Mean ± SD	105.58 ± 20.54	104.21 ± 17.55	108.13 ± 25.71	0.56
**Respiratory rate**				
Mean ± SD	20.19 ± 2.52	20.00 ± 2.40	20.53 ± 2.80	0.51
**SpO2**				
Mean ± SD	90.49 ± 7.57	91.71 ± 6.45	88.20 ± 9.12	0.15
**Body mass index (BMI)**				
Mean ± SD	27.12 ± 5.38	28.96 ± 4.30	23.57 ± 5.70	0.001

SD, standard deviation; FATA, Federally Administered Tribal Area; SpO2, blood oxygen saturation.

**Table 2:** Laboratory and Radiological findings of cancer patients with diabetes and COVID-19.

	Total (n = 43)	Alive 28 (65.1%)	Dead 15 (34.9%)	*P*-value
**White blood cells, ×** 10^3^ cells/µl				0.25
Median (range)	7.00 (1-197)	7 (1-197)	10 (1-24)	
**Neutrophils, ×** 10^3^ cells/µl				0.04
Median (range)	6.00 (1-22)	4.50 (1-21.21)	8.00 (1-22)	
**Lymphocytes, ×** 10^3^ cells/µL				0.51
Median (range)	1.00 (1-184)	1.00 (1-184)	1.00 (1-4.00)	
**Platelets, ×** 10^3^ cells/µL				0.65
Median (range)	209.50 (14-446)	221.50 (73-423)	198.50 (14-446)	
**RBCs, ×** 10^6^ cells/µL				0.24
Median(range)	4.00 (2-6)	4.00 (2-6)	4 (2-5)	
**Hemoglobin,** g/dL				0.08
Median (range)	10.40 (6.00-14.80)	10.70 (6.00-14.80)	9.30 (7.30-12.60)	
**Creatinine,** mg/dL				0.25
Median (range)	1.00 (1.00-5.00)	1.00 (1.00-5.00)	1.00 (1.00-5.00)	
**Blood urea nitrogen,** mg/dL				0.38
Median (range)	17.50 (4.00-93.00)	16.00 (6.00-42.00)	18.50 (4.00-93.00)	
**Lactate dehydrogenase,** u/L				0.65
Median(range)	326.50 (219-579)	366.60 (219-579)	253 (231-442)	
**Lactate,** mg/dL				0.01
Median (range)	16 (7-153)	11 (7-23)	20 (9-153)	
**D-dimer,** mg/L				0.46
Median (range)	2 (1-80)	1.50 (1-5)	3 (1-80)	
<0.2	1 (2.3)	1 (3.6)	–	0.72
≥0.2	12 (27.9)	9 (32.1)	3 (20.0)	
**pH**				0.72
Median(range)	7 (7-8)	7 (7-8)	7 (7-8)	
**PCO2,** mmHg				0.83
Median(range)	3 (1-9)	3 (1-4)	3 (1-9)	
**PO2,** mmHg				0.73
Median(range)	63 (10-95)	63 (15-84)	61 (10-95)	
**HCO3,** mmol/L				0.94
Median(range)	22 (4-57)	21.50 (15-28)	24 (4-57)	
**O2 saturation,** %				1.00
Median(range)	93 (10-99)	93 (10-99)	92 (10-98)	
**C-reactive protein,** mg/L				0.005
Median(range)	146.50 (17-407)	91 (17-372)	173 (109-407)	
**ALT,** U/L				0.32
Median(range)	22 (7-91)	23 (9-91)	18 (7-67)	
**AST,** U/L				0.88
Median(range)	42 (11-752)	42 (11-176)	41 (19-752)	
**Albumin,** g/dL				0.02
Median (range)	3.00 (2.00-4.00)	3 (2-4)	2 (2-4)	
**Globulin,** g/dL				0.75
Median(range)	3.00 (1.00-5.00)	3 (1-5)	3 (2-4)	
**GGT,** U/L				0.10
Median(range)	59 (10-869)	51 (10-223)	119 (22-869)	
**Alkaline phosphate,** U/L				0.03
Median(range)	101 (50-487)	90 (50-203)	170 (63-487)	
**Ferritin,** ng/mL				0.51
Median(range)	299 (78-970)	303 (110-802)	243 (78-970)	
**HbA1c**				0.49
Median(range)	8.5 (6-14)	9 (6-14)	8 (6-12)	
**Blood glucose level**				0.13
Median(range)	220.50 (102-544)	248 (102-544)	175 (109-359)	
**Radiological findings**				0.56
Normal	6 (14.0)	3 (10.7)	3 (20.0)	
Consolidation	20 (46.5)	12 (42.9)	8 (53.3)	
Patchy shadowing	8 (18.6)	7 (25.0)	1 (6.7)	
Reticulonodular infiltrates	1 (2.3)	1 (3.6)	–	
Unknown	8 (18.6)	5 (17.9)	3 (20.0)	

RBCs, red blood cells; ALT, alanine transaminase; AST, aspartate transaminase; HbA1c, Glycated hemoglobin; GGT, gamma-glutamyl transpeptidase.

Of the 43 patients included, 40 (93.0%) received intravenous antibiotics and 10 (23.3%) received antiviral medication (**Table 3**). 37 (86.0%) of 43 were on antidiabetic medication (**Supplementary Data Table 1**). Oxygen therapy was given to 33 (76.7%) patients. ACE/ARBs were given to 10 (23.3%) patients (**Table 3**). Invasive and non-invasive mechanical ventilation was provided to 6 (14.0%) and 9 (20.9%) patients, respectively. 11 (25.6%) of 43 patients were admitted to the ICU. 36(83.7%) of 43 patients developed the complications such as ARDS 22 (51.2%), acute renal failure 12 (27.9%), septic shock 8 (18.6%), abnormal liver function 10 (23.3%), coagulopathy 24 (55.8%), secondary infections 23 (53.5%) and arrhythmia 2 (4.7%). Moreover, all the deceased patients (n=15) had acquired the complications (**Table 3**).

**Table 3:** Treatments and complications of cancer patients with diabetes and COVID-19.

	Total (n = 43)	Alive 28 (65.1%)	Dead 15 (34.9%)	*P*-value
**Treatments**				
Intravenous antibiotic	40 (93.0)	25 (89.3)	15 (100.0)	0.54
Antiviral medication	10 (23.3)	8 (28.6)	2 (13.3)	0.45
Montelukast	11 (25.6)	8 (28.6)	3 (20.0)	0.72
Zinc (vitamin C)	10 (23.3)	8 (28.6)	2 (13.3)	0.45
Oxygen therapy	33 (76.7)	19 (67.9)	14 (93.3)	0.13
ACE/ARBs	10 (23.3)	7 (25.0)	3 (20.0)	1.00
**Mechanical ventilator**				0.13
None	28 (65.1)	21 (75.0)	7 (46.7)	
Non-invasive	9 (20.9)	5 (17.9)	4 (26.7)	
Invasive	6 (14.0)	2 (7.1)	4 (26.7)	
**Admission to ICU**	11 (25.6)	5 (17.9)	6 (40.0)	0.15
CRRT	2 (4.7)	1 (3.6)	1 (6.7)	1.00
**Complications**	36 (83.7)	21 (75.0)	15 (100)	0.07
ARDS	22 (51.2)	9 (32.1)	13 (86.7)	0.001
Acute renal failure	12 (27.9)	6 (21.4)	6 (40.0)	0.30
Septic shock	8 (18.6)	2 (7.1)	6 (40.0)	0.01
Abnormal liver function	10 (23.3)	3 (10.7)	7 (46.7)	0.001
Coagulopathy	24 (55.8)	16 (57.1)	8 (53.4)	0.001
Secondary infection	23 (53.5)	11 (39.3)	12 (80.0)	0.023
Arrhythmia	2 (4.7)	–	2 (13.3)	0.12

ACE/ARBs, angiotensin converting enzyme inhibitors and angiotensin II receptor blockers; ICU, Intensive-care unit; CRRT, continuous renal replacement therapy; ARDS, acute respiratory distress syndrome.

39 (90.7%) of 43 patients were diagnosed with solid tumors and 3 (7.0%) of 43 patients were diagnosed with hematological malignancies. Additionally, 1 (2.3) patient had both solid and hematological malignancy (**Table 4**). The most common cancers were breast cancer 10 (25.6%) and prostate cancer 5 (12.8%). Among hematological malignancies, there were three types, chronic lymphocytic leukemia 1(33.3%), Burkitt’s lymphoma 1(33.3%), and multiple myeloma 1(33.3%). One had both renal cell carcinoma and chronic lymphocytic leukemia 1 (2.3%). 23 (53.5%) of 43 patients received chemotherapy. Radiotherapy was given to 19 (44.2%) of 43 patients. 27 (62.8%) patients underwent the surgery. 9 (20.9%) of 43 patients received chemotherapy within 4 weeks before the onset of symptoms.

**Table 4:** History of cancer in diabetic patients with COVID-19.

	Total (n = 43)	Alive 28 (65.1%)	Dead 15 (34.9%)	*P*-value
**Cancer Type**				
**Both (solid + hematological)**	1 (2.3)	1 (100.0)	–	1.00
Renal cell carcinoma + Chronic lymphocytic leukemia	1 (100.0)	1 (100.0)	–	
**Hematological malignancy**	3 (7.0)	1 (33.3)	2 (66.7)	0.22
Chronic lymphocytic leukemia	1 (33.3)	1 (100.0)	–	
Burkitt’s lymphoma	1 (33.3)	–	1 (100.0)	
Multiple myeloma	1 (33.3)	–	1 (50.0)	
**Solid malignancy**	39 (90.7)	26 (66.7)	13 (33.3)	0.13
Breast	10 (25.6)	6 (23.1)	4 (30.8)	
Bladder	4 (10.3)	4 (15.4)	–	
Prostate	5 (12.8)	3 (11.5)	2 (15.4)	
Kidney	4 (10.3)	4 (15.4)	–	
Pancreas	3 (7.7)	1 (3.8)	2 (15.4)	
Others	13 (33.3)	8 (30.8)	5 (38.5)	
**History of treatment All**	38 (88.4)	25 (89.3)	13 (86.7)	1.00
Surgery	27 (62.8)	21 (75.0)	6 (40.0)	0.04
Chemotherapy	23 (53.5)	14 (50.0)	9 (60.0)	0.53
Radiotherapy	19 (44.2)	14 (50.0)	5 (33.3)	0.29
**Chemotherapy within 4 weeks before symptoms onset**				
Chemotherapy	9 (20.9)	6 (21.4)	3 (20.0)	1.00
**Stage**				0.97
Early (I-II)	19 (44.2)	13 (46.4)	6 (40.0)	
Advance (III-IV)	24 (55.9)	15 (53.5)	9 (60.0)	
**Time since cancer diagnosis**				0.13
<1	4 (9.3)	1 (3.6)	3 (20.0)	
1-5 years	28 (65.1)	18 (64.3)	10 (66.7)	
>5	11 (25.6)	9 (32.1)	2 (13.3)	
**ECOG performance status score**				0.55
0	5 (11.6)	4 (14.3)	1 (6.7)	
1	12 (27.9)	8 (28.6)	4 (26.7)	
2	19 (44.2)	13 (46.4)	6 (40.0)	
3	3 (7.0)	2 (7.1)	1 (6.7)	
4	4 (9.3)	1 (3.6)	3 (20.0)	

ECOG, Eastern Cooperative Oncology Group.

**Figure 2:**
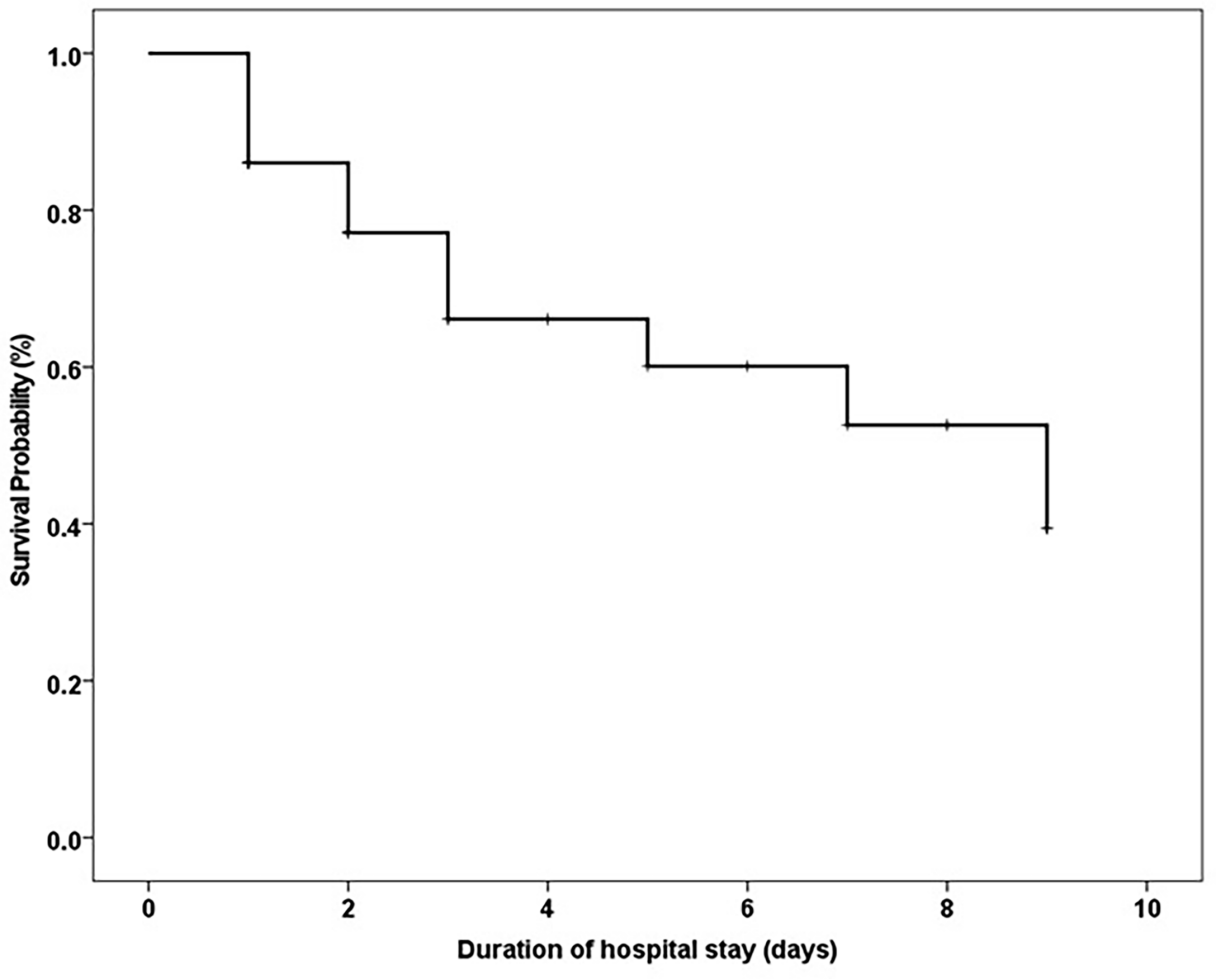
Overall Survival of COVID-19 cancer patients with diabetes.

4 (9.3%) of 43 patients were diagnosed with cancer within the past year. 28 (65.1%) of 43 patients were diagnosed with cancer within the past year five years and 26 (60.4%) of 43 patients had an ECOG score higher than one before admission. 24 (55.9%) of 43 patients were in an advanced stage (III-IV). The case fatality rate in patients with solid tumors was 33.3% (13 of 39 patients) and that in hematological malignancies was 66.6% (2 of 3 patients), (**Table 4**). The median time of hospital stay was 2 days (range 1-9 days). The overall median survival time of hospital stay was 9 days (confidence interval 4-14 days) **Figure 2**.

## Discussion

Cancer patients are highly susceptible individuals in the COVID-19 pandemic (14, 19–21). It has been reported that the mortality rate is high in these patients (14). Diabetes is one of the leading causes of morbidity and mortality worldwide (22). This disorder is associated with several complications that eventually influence the overall survival of patients (23). It has been previously identified that there is an association between diabetes and infection (24). However, the data remains contentious regarding whether diabetes itself augments vulnerability and influences outcomes from infections (25). The scientific data on COVID-19-infected cancer patients with diabetes is scarce. This is the first retrospective study conducted in Pakistan to demonstrate the clinical features and management of COVID-19-infected cancer patients with diabetes.

COVID-19 disease characteristically showed two clinical phases (26). During the first phase (5 days after infection), patients undergo extensive viral replication (26). During this phase, the symptoms are mostly fever and dyspnea (26–29). In the current study, we observed that fever (74.4%) and dyspnea (58.1%) were the most common symptoms as well. After the onset of the symptoms (7-10 days), some patients may enter into a second phase (26). During this phase, patients develop pathological changes due to an explicit immune response caused by the release of cytokine storm (26). The patients eventually become seriously ill and may require ICU admission (27, 29).

In the current pandemic, some studies did not observe a well-defined relationship between diabetes and severe disease (22, 30). Nevertheless, few studies showed that elderly diabetic patients with comorbidities were at higher risk to develop severe COVID-19 and mortality (6, 7, 31). Although in our data, the overall mean age was 61.67 ± 11.80, we could not find a significant difference in ages between survivors and non-survivors COVID-19 infected cancer patients with diabetes. Furthermore, we observed that cardiovascular and renal comorbidities, including hypertension, were more frequent in our data set. We also observed abnormal pulse rate and blood oxygen saturation in the COVID-19-infected cancer patients with diabetes. Xie et al. reported a link between hypoxemia (SpO2 <90%) and mortality in COVID-19 patients (32). We found lower levels of blood oxygen saturation (SpO2) in non-survivors patients. Yang et al. also observed lower levels of blood oxygen saturation among non-survivors (14). Overweight and obesity are linked with poor prognosis in COVID-19 infected diabetic patients (33). We observed a statistically significant mean difference in body mass index (BMI) between survivors and non-survivors (P=0.001). Additionally, in non- survivor patients 4 (27%) had underweight BMI. Ye et al. reported that both underweight and overweight COVID-19 infected patients have a tendency to acquire acute lung injury as compared with normal-weight patients. Besides that, especially underweight patients were more prone to develop a secondary infection (34). In our data set, all the underweight patients were non-survivor and they acquired the secondary infection as well.

Pro-inflammatory neutrophils and C-reactive protein (CRP) can be used as prognostic indicators for critical illness and adverse clinical outcomes in the patients infected with COVID-19 (35). Zhang et al. reported higher levels of C-reactive protein and neutrophil counts in cancer patients infected with COVID-19 (35). We also observed higher levels of C-reactive protein and neutrophil counts in non-survivor *vs* survivor COVID-19 infected cancer patients with diabetes. There is a possibility that pro-inflammatory neutrophils and C-reactive protein provoke “cytokine storm” associated to endothelialitis in COVID-19 infected cancer patients (35, 36). Zhou et al. confirmed that SARS-CoV-2 utilizes angiotensin-converting enzyme 2 (ACE2) receptor to gain entry into cells (37). Hamming et al. observed that ACE2 receptors are not only expressed in alveolar epithelial cells but also in cells of bile duct (38). These studies propose that SARS-CoV-2 may infect the bile duct cells and cause abnormal liver function in these patients. Nevertheless, alkaline phosphatase (ALP) which is the bile duct injury marker, has been reported as not elevated in COVID-19 infected patients (1). Recently published data by Kumar et al. concluded that ALP is a better indicator of COVID-19 induced liver injury (39). We also found a statistically significant (*P*=0.03) difference of ALP in survivor and non-survivor COVID-19 infected cancer patients with diabetes. Hyperlactatemia may be considered as a predictor of death (40). Evaluation of serum lactate has clinical implication in the diagnosis and treatment of serious cases of severe sepsis (41). We observed a significant increase in the lactate levels of non-survivor COVID-19 infected cancer patients with diabetes. COVID-19 infected patients with hypoalbuminemia have a tendency to develop severe clinical manifestations (42). Huang et al. identified a strong association of systemic inflammation in COVID-19 with hypoalbuminemia (43). They also observed a considerable difference in albumin between survivors and non‐survivors COVID-19 infected patients (43). We found similar results in our study. Therefore, screening of neutrophil counts, CRP, ALP, serum lactate and albumin levels is advised in diabetic cancer patients infected with SARS-CoV-2.

Diabetic patients are at an increased risk of developing the severe form of COVID-19 (44). Salehi et al. reported that the finding of consolidation was the initial presentation among COVID-19 infected patients (45). In the current study, 67.4% patients showed abnormal features in the radiological findings and 46.5% patients presented with consolidation. The involvement of the lungs indicates that cancer patients with diabetes are more prone to serious lung disease after COVID-19-infection (11).

Furthermore, we observed that complications such as ARDS, septic shock, coagulopathy, abnormal liver function and secondary infection were common in these patients. Our findings are in keeping with the data published previously (14). Yang et al. described that COVID-19 infected cancer patients receiving chemotherapy within four weeks before the onset of symptoms were vulnerable to death (14). In contrast, another study on COVID-19 infected cancer patients demonstrated that chemotherapy had no effect on patient death (42). We observed a similar finding in COVID-19 infected cancer patients with diabetes.

We acknowledge some limitations. Our current study is a retrospective cohort. We enrolled only those diabetic cancer patients who were infected with COVID-19 infection and admitted to our hospital from March 2020 to May 18, 2021. The sample size of our study was limited because of a small population of diabetic cancer patients who tested positive for COVID-19 infection. Additionally, we have not assessed the variation in pathological factors during hospitalization. To characterize the effect of the disease, a long-term follow up of the COVID-19 infected survivor diabetic cancer patients is required. Another limitation of our study is that since our hospital is a dedicated cancer care hospital, we could not include non-cancer patients.

Despite these limitations, the study had substantial strengths. Scarce data exist regarding clinical characteristics of survivors and non-survivors COVID-19-infected diabetic cancer patients. It is an attempt to identify the risk factors associated with severe disease in this highly susceptible group of patients in a pandemic. Besides that, it is the first study conducted in Pakistan for this specific cohort of patients.

In conclusion, we presented comprehensive data of the clinical features and outcomes of COVID-19-infected cancer patients with diabetes from Pakistan. Although diabetes is linked with severe outcomes in COVID-19 patients, the diabetic patients may not be highly susceptible to SARS-CoV-2 infection (16, 46–50). Since severity of COVID-19 changes promptly, it is favorable to identify the contributors of severity at the initial stage. Diabetes along with other comorbidities are substantial predictors of mortality in COVID-19 patients. We need to be more alert to the levels of CRP, ALP, serum lactate, albumin, neutrophils and regular monitoring of blood glucose, once diabetic cancer patients are infected with COVID-19. Future studies are warranted to understand the pathophysiological mechanisms of the association between COVID-19 and diabetes.

## Data availability statement

The original contributions presented in the study are included in the article/**Supplementary Material**. Further inquiries can be directed to the corresponding author.

## Ethics statement

The studies involving human participants were reviewed and approved by institutional review board (IRB) of SKMCH & RC. Written informed consent for participation was not required for this study in accordance with the national legislation and the institutional requirements.

## Author contributions

KA had the idea for and designed the study. KA, MA, SA, AA, AF, and RR were involved in the acquisition of the data. KA, MA, AF, and RR summarized the data. KA, MA, WS, UA, AS, and KS were involved in data interpretation. KA and MA drafted the manuscript. MA, KS, UA, AS, and WS critically revised the manuscript for important intellectual content. All authors contributed to the article and approved the submitted version.

## Acknowledgments

We would like to appreciate all hospital management and healthcare professionals who worked extensively during the pandemic.

## Conflict of interest

The authors declare that the research was conducted in the absence of any commercial or financial relationships that could be construed as a potential conflict of interest.

## Publisher’s note

All claims expressed in this article are solely those of the authors and do not necessarily represent those of their affiliated organizations, or those of the publisher, the editors and the reviewers. Any product that may be evaluated in this article, or claim that may be made by its manufacturer, is not guaranteed or endorsed by the publisher.
