# Supplementary material for: COVID-19 in cancer patients with diabetes in Pakistan: Clinical features and management
Source: Front Oncol. 2022 Aug 18;12:922579. doi: 10.3389/fonc.2022.922579 (PMC9434633; doi:10.3389/fonc.2022.922579)
Supplement: Supplementary file 1 [file Table_1.docx]

**Table 1: Status of antidiabetic medication in cancer patients with diabetes and COVID-19**

| **Patient ID** | **Diabetic medication** |
| --- | --- |
| P-01 | Oral Hypoglycemics + Insulin |
| P-02 | Diet |
| P-03 | Oral Hypoglycemics |
| P-04 | Oral Hypoglycemics |
| P-05 | Oral Hypoglycemics |
| P-06 | Oral Hypoglycemics + Insulin |
| P-07 | Diet |
| P-08 | Oral Hypoglycemics |
| P-09 | Oral Hypoglycemics |
| P-10 | Oral Hypoglycemics + Insulin |
| P-11 | Insulin |
| P-12 | Oral Hypoglycemics |
| P-13 | Insulin |
| P-14 | Insulin |
| P-15 | Oral Hypoglycemics + Insulin |
| P-16 | Oral Hypoglycemics |
| P-17 | Oral Hypoglycemics + Insulin |
| P-18 | Diet |
| P-19 | Oral Hypoglycemics |
| P-20 | Insulin |
| P-21 | Diet |
| P-22 | Oral Hypoglycemics + Insulin |
| P-23 | Insulin |
| P-24 | Diet |
| P-25 | Insulin |
| P-26 | Oral Hypoglycemics |
| P-27 | Oral Hypoglycemics + Insulin |
| P-28 | Oral Hypoglycemics |
| P-29 | Insulin |
| P-30 | Oral Hypoglycemics |
| P-31 | Insulin |
| P-32 | Insulin |
| P-33 | Oral Hypoglycemics + Insulin |
| P-34 | Oral Hypoglycemics |
| P-35 | Insulin |
| P-36 | Diet |
| P-37 | Insulin |
| P-38 | Oral Hypoglycemics |
| P-39 | Oral Hypoglycemics |
| P-40 | Insulin |
| P-41 | Oral Hypoglycemics + Insulin |
| P-42 | Oral Hypoglycemics |
| P-43 | Oral Hypoglycemics + Insulin |
